# Two distinct trajectories of brain volume loss in myotonic dystrophy type 1 via machine learning

**DOI:** 10.1093/braincomms/fcaf181

**Published:** 2025-05-07

**Authors:** Tomoki Imokawa, Hiroyuki Maki, Daichi Sone, Risa Kagaya, Yoko Shigemoto, Yukio Kimura, Hiroshi Matsuda, Yuji Takahashi, Ukihide Tateishi, Noriko Sato

**Affiliations:** Department of Radiology, National Centre Hospital, National Centre of Neurology and Psychiatry, 187-8551 Kodaira, Tokyo, Japan; Department of Diagnostic Radiology, Institute of Science Tokyo, 113-8510 Bunkyo-ku, Tokyo, Japan; Department of Radiology, National Centre Hospital, National Centre of Neurology and Psychiatry, 187-8551 Kodaira, Tokyo, Japan; Department of Psychiatry, Jikei University School of Medicine, 105-0003 Minato-ku, Tokyo, Japan; Department of Radiology, National Centre Hospital, National Centre of Neurology and Psychiatry, 187-8551 Kodaira, Tokyo, Japan; Department of Radiology, National Centre Hospital, National Centre of Neurology and Psychiatry, 187-8551 Kodaira, Tokyo, Japan; Department of Radiology, National Centre Hospital, National Centre of Neurology and Psychiatry, 187-8551 Kodaira, Tokyo, Japan; Department of Biofunctional Imaging, Fukushima Medical University, 960-1295 Fukushima-shi, Fukushima, Japan; Department of Neurology, National Centre Hospital, National Centre of Neurology and Psychiatry, 187-8551 Kodaira, Tokyo, Japan; Department of Diagnostic Radiology, Institute of Science Tokyo, 113-8510 Bunkyo-ku, Tokyo, Japan; Department of Radiology, National Centre Hospital, National Centre of Neurology and Psychiatry, 187-8551 Kodaira, Tokyo, Japan

**Keywords:** SuStaIn, muscular dystrophy, neurodegenerative disease, tauopathy, dementia

## Abstract

Myotonic dystrophy Type 1 is a disorder that affects multiple systems, including the muscles and the CNS. Previous studies have primarily used voxel-based morphometry to examine areas of brain volume reduction and their correlation with symptoms; however, consistent findings have not been obtained. Subtype and stage inference is an unsupervised machine learning algorithm that elucidates disease progression and subtypes from cross-sectional data. In this study, we used Subtype and Stage Inference to analyse the morphometric MRI data of patients with myotonic dystrophy Type 1 to reveal the detailed trajectories of brain volume loss and to explore the potential of morphometric MRI as a biomarker for myotonic dystrophy Type 1. We examined 60 patients with myotonic dystrophy Type 1 and 50 age- and sex-matched controls. The patients with myotonic dystrophy Type 1 had a median age of 44 years (range 20–67 years) and included 32 males. Using three-dimensional T1-weighted MRI images, we analysed the subtypes of brain involvement and their respective trajectories of brain volume loss with subtype and stage inference. Additionally, we examined the differences and correlations in clinical and brain morphological indicators between the identified subtypes and controls. Subtype and stage inference revealed two subtypes: cortical and subcortical. In the cortical subtype, volume reduction began in the precentral gyrus and spread primarily to the cerebral cortex. In the subcortical subtype, it progressed early in the putamen, thalamus, hippocampus and amygdala. Examination of clinical indicators showed that despite the younger age of the subcortical subtype compared to the cortical subtype, mini-mental state examination scores were significantly lower in the subcortical subtype and negatively correlated with subcortical probability. The total intracranial volume, a marker of maximal brain growth, was significantly smaller in the cortical subtype; however, it was not smaller in the subcortical subtype than in controls. Furthermore, the subcortical subtype showed a larger total ventricle volume than both the controls and the cortical subtype. In contrast, its total brain parenchymal volume was lower than that of the controls, similar to the cortical subtype. These results suggest early childhood brain development differences between the two subtypes. Using Subtype and Stage Inference, we identified two subtypes of myotonic dystrophy Type 1 and demonstrated the potential of morphological MRI as a biomarker for cognitive impairment and brain developmental disorders. Machine learning can aid in stratifying myotonic dystrophy Type 1 in clinical settings and contribute to the elucidation of its complex pathophysiology.

## Teaser

Linking brain imaging findings to the symptoms of myotonic dystrophy Type 1 is challenging. Imokawa *et al*. used machine learning to identify two brain volume loss trajectories, cortical and subcortical, with the latter linked to cognitive impairment. This study demonstrated the potential of MRI as an indicator of cognitive decline.

## Graphical Abstract

**Graphical Abstract:**
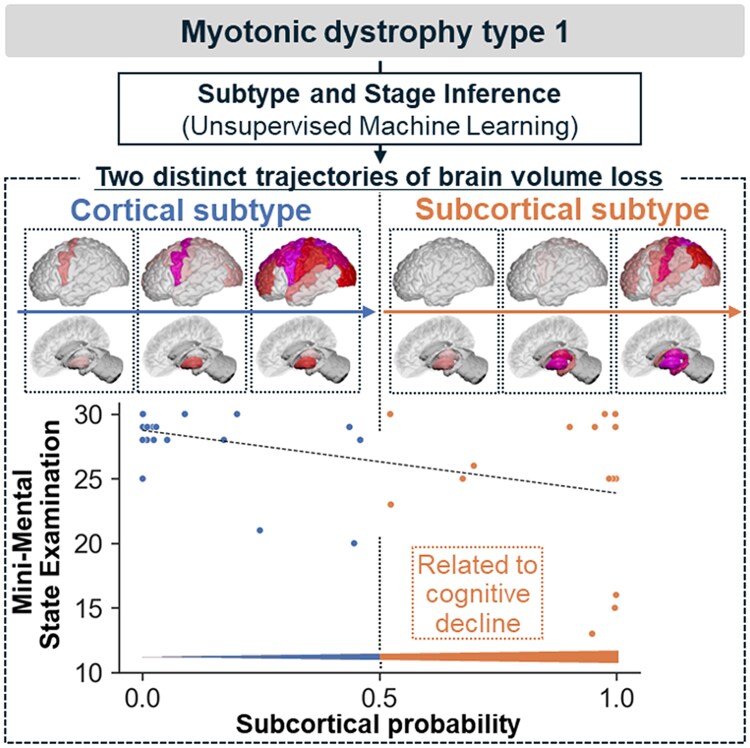

## Introduction

Myotonic dystrophy Type 1 is an autosomal dominant form of muscular dystrophy caused by a CTG repeat expansion in the untranslated 3′ region of myotonic dystrophy protein kinase (*DMPK*) on chromosome 19q13.^1^ Myotonic dystrophy Type 1 affects multiple organs, including the CNS. Pathological studies of the CNS suggest that myotonic dystrophy Type 1 involves a combination of RNAopathy, spliceopathy and tauopathy,^2^ leading to cognitive decline beyond normal ageing in domains such as memory, attention and visuospatial/visuoconstructive abilities.^3-7^ Additionally, brain developmental abnormalities have also been speculated in adult-onset myotonic dystrophy Type 1 based on the reports indicating reduced total intracranial volume (TIV), which reflects maximal brain growth.^8,9^ Despite being the most common adult-onset muscular dystrophy worldwide, affecting at least 1 in 8000 individuals,^10^ disease-modifying treatments are currently unavailable. Understanding the CNS pathophysiology of myotonic dystrophy Type 1 may help identify therapeutic targets.

Previous studies using voxel-based morphometry (VBM) to elucidate brain structural changes in myotonic dystrophy Type 1 have shown volume reductions in areas such as the frontal and parietal lobes (including the region around the central sulcus), temporal lobe, hippocampus, lenticular nucleus and thalamus.^11-13^ Several longitudinal studies have been conducted to examine changes in brain volume over time; however, these involved relatively small patient groups of 13–21 individuals and had limited follow-up periods of 5.5–13.4 years, resulting in inconsistent findings.^14-16^ Additionally, while some studies have demonstrated correlations between brain structural changes and neuropsychological or clinical parameters,^17-21^ others have not,^8,22-24^ leaving the role of MRI as a biomarker for myotonic dystrophy Type 1 uncertain.

Subtype and Stage Inference (SuStaIn) is an unsupervised machine learning method that can identify disease subtypes and temporal progression patterns from cross-sectional data, without the need for longitudinal data.^25^ SuStaIn can be applied to various data expressed in Z-scores. It has been utilized in neurodegenerative diseases to analyse brain volume changes in T1-weighted images, white matter degeneration in diffusion tensor images and amyloid or tau accumulation in PET scans.^26-30^ However, studies employing SuStaIn for myotonic dystrophy Type 1 are lacking. The inconsistency in previous VBM studies may be due to unknown disease progression patterns and subtypes. The SuStaIn analysis may reveal these hidden patterns.

In this study, we used SuStaIn to analyse the morphometric MRI data of patients with myotonic dystrophy Type 1, to enhance our understanding of brain pathology. To examine potential differences in childhood trajectories of the CNS, we also analysed morphometric indicators including TIV. Furthermore, by examining the relationships between each patient’s subtype and stage as determined by SuStaIn and their clinical indicators, we explored the potential of MRI as a biomarker for myotonic dystrophy Type 1.

## Materials and methods

### Participants

This retrospective study was approved by the hospital’s Institutional Review Board, which performed the clinical and imaging studies. Seventy patients with myotonic dystrophy Type 1 who visited our hospital and underwent MRI between January 2018 and December 2023 were enrolled in the study. The diagnosis of myotonic dystrophy Type 1 was confirmed by identifying ≥50 CTG repeats in the *DMPK* gene. Five patients were excluded due to a lack of appropriate MRI data, two due to missing information on the CTG repeat length, and three due to lesions of another aetiology (two with meningiomas and one with brain contusion). Therefore, 60 patients with myotonic dystrophy Type 1 were included. Medical records were reviewed for age, onset age, CTG repeat expansion in the *DMPK* gene, muscular impairment rating scale (MIRS) score and mini-mental state examination (MMSE) score. The MIRS is a five-point scale that evaluates muscle involvement in patients with myotonic dystrophy Type 1. Data on CTG repeat length and MIRS scores were available for all individuals in the myotonic dystrophy Type 1 group, whereas MMSE data were available for 40 individuals. In addition, 50 age- and sex-matched healthy controls were recruited based on the following criteria: no history of neurological diseases, mental health issues, or use of medications affecting the CNS. Written informed consent was obtained from each control participant.

### MRI data acquisition and pre-processing

All participants were scanned using a 3T clinical scanner (Achieva; Philips Healthcare, Netherlands) with a 32-channel head coil. Three-dimensional (3D) T1-weighted images were acquired in the sagittal plane. The conditions were as follows: repetition time/echo time, 7.18/3.46 ms; inversion time 1100 ms, flip angle, 10°; voxel size = 0.68 × 0.68 × 0.6 mm^3^; matrix, 384 × 384; field-of-view, 260 × 260 mm; number of excitations, 1.

Using FreeSurfer 7.4 (http://surfer.nmr.mgh.harvard.edu/), we segmented the 3D T1-weighted images of all participants. We measured the cortical thickness and subcortical grey matter volume for each region, TIV, total ventricle volume and total brain parenchymal volume. The total ventricle volume was calculated as the sum of the volumes of the bilateral lateral ventricles, third ventricle and fourth ventricle obtained using FreeSurfer. We used the ‘BrainSegVolNotVent’ value provided by FreeSurfer as total brain parenchymal volume. Cortical segmentation was based on the Desikan–Killiany Atlas,^31^ whereas subcortical segmentation was based on an atlas containing probabilistic information on the location of structures.^32^ Segmentation accuracy was confirmed for all participants through visual inspection. In addition to visual inspection, we assessed the quality of the cortical surface reconstructions using the Euler number calculated by FreeSurfer. The Euler number, which quantifies the topology of the reconstructed cortical surface, was computed for each hemisphere and the average value across the left and right hemispheres was reported for each participant. For a completely flat and smooth surface, the Euler number should be two. For a surface containing defects, the Euler number is calculated as 2–2*n*, where *n* is the number of defects. The median Euler number across participants was −46 (range −204–−16), with individual values falling within the acceptable range for cortical surface reconstruction.^33^  Supplementary Fig. 1 illustrates the segmentation results with the highest and lowest Euler numbers.

Based on previous VBM studies,^11-13^ we selected 20 regions of interest (ROIs): the caudal middle frontal, rostral middle frontal, superior frontal, precentral, postcentral, superior parietal, inferior parietal, supramarginal, superior temporal, middle temporal, inferior temporal, transverse temporal, lateral occipital, insula, thalamus, caudate, putamen, pallidum, hippocampus and amygdala (Supplementary Fig. 2). We used volume for the subcortical and mesiotemporal ROIs, whereas we used thickness instead of volume for the cortical ROIs. This approach is consistent with previous SuStaIn studies.^34,35^ Using cortical thickness allows us to avoid the confounding effects of surface area and TIV on cortical volume and may provide a more accurate reflection of neurodegeneration.^36-38^ Since no asymmetry in brain atrophy was observed in myotonic dystrophy Type 1, the thickness or volume of the left and right sides of each region was summed.^11,13^ Following prior research using the SuStaIn algorithm on MRI data, we adjusted cortical thickness for age and sex (binary) and the subcortical and mesiotemporal volumes for age, sex and TIV to calculate Z-scores.^34,35^ Specifically, we first performed a linear regression using the control group data, with the aforementioned variables as independent variables and each MRI measure as the dependent variable. Our control group of 50 participants was sufficient for linear regression with up to three variables, exceeding the recommended minimum of 10 participants per variable.^39^ Residuals from this regression analysis were obtained for all participants, including both the control and myotonic dystrophy Type 1 groups. The residuals for each region were expressed as Z-scores relative to the control group, ensuring that the control group had a mean of 0 and a standard deviation of 1. In the SuStaIn model, the Z-scores were defined as increasing with stage progression. In myotonic dystrophy Type 1, as in other neurodegenerative diseases, the cerebral cortex and subcortical grey matter progressively atrophied over time.^40^ Therefore, the Z-scores were multiplied by −1 so that a lower thickness or volume corresponded to larger positive Z-scores.

### SuStaIn

In this study, we used SuStaIn to elucidate the temporal and spatial progression patterns of brain involvement in patients with myotonic dystrophy Type 1 using cross-sectional imaging data.^25,41^ SuStaIn is an unsupervised machine learning method that simultaneously clusters individuals into groups (subtypes) and reconstructs disease progression patterns for each group without relying on prior assumptions. The disease progression pattern for each subtype was represented as a sequence of stages, with each stage corresponding to a biomarker (thickness or volume of a brain region) reaching a new Z-score. For each biomarker, the Z-score was modelled to increase linearly in a stepwise manner through several predefined values, ultimately reaching the maximum Z-score (Z-max) at the final stage. Model fitting was performed by incrementally increasing the number of subtypes, starting from one. Using a greedy optimization approach, the set of sequences and the proportion of each subtype (f) were determined to maximize the likelihood of the given cross-sectional data for each specified number of subtypes. For a detailed formalization and mathematical modelling of SuStaIn, see a previous publication.^25^

**Figure 1:**
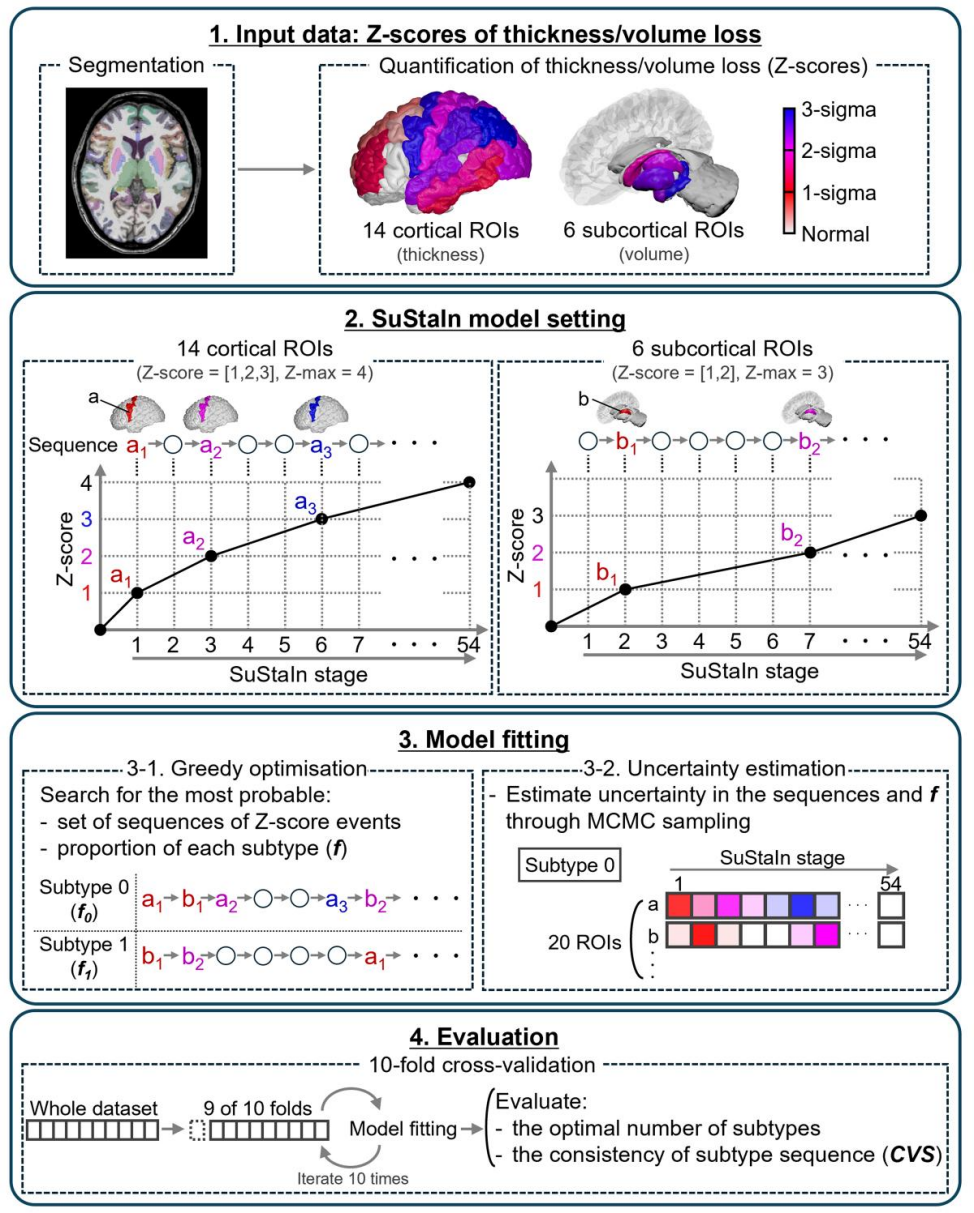
**The pipeline of SuStaIn analysis.** First, the input data were prepared. Using FreeSurfer 7.4, we segmented 3D T1-weighted images for all participants (60 patients with myotonic dystrophy Type 1 and 50 age- and sex-matched healthy controls). The thicknesses of 14 cortical ROIs and the volumes of 6 subcortical ROIs were then expressed as Z-scores relative to the control group, ensuring a mean of 0 and a standard deviation of 1 for the control group. The Z-scores were multiplied by −1 so that lower thickness or volume corresponded to larger positive Z-scores. Secondly, the SuStaIn model was set up. SuStaIn is an unsupervised machine learning method that simultaneously clusters individuals into groups (subtypes) and reconstructs a disease progression pattern for each group, without relying on prior assumptions. The disease progression pattern for each subtype is represented as a sequence of stages, with each stage corresponding to a biomarker (thickness or volume of a brain region) reaching a new Z-score. For each biomarker, the Z-score is modelled to increase linearly in a stepwise manner through several predefined values, ultimately reaching Z-max at the final stage. For the 14 cortical ROIs based on thickness, the Z-score steps were set to 1, 2 and 3, with a Z-max of 4. For the six ROIs based on volumes in mesiotemporal and subcortical areas, the Z-score steps were set to 1 and 2, with a Z-max of 3. ROI a and ROI b are examples of cortical and subcortical ROIs, respectively. For instance, a_1_ represents the event of ROI a reaching a Z-score of 1. Thirdly, model fitting was performed by incrementally increasing the number of subtypes, from one to three. The set of sequences and the proportion of each subtype (**F**) were determined to maximize the likelihood of the given cross-sectional data for each specified number of subtypes, using a greedy optimization approach. Subsequently, uncertainty estimation was conducted using 1 000 000 MCMC samples initialized from the maximum likelihood solution. Fourthly, 10-fold cross-validation was conducted to evaluate the optimal number of subtypes. The consistency of subtype sequences was assessed using CVS. CVS, cross-validation similarity; MCMC, Markov Chain Monte Carlo; ROI, region of interest; SuStaIn, subtype and stage inference; Z-max, the maximum Z-score.

We analysed the Z-scores of the myotonic dystrophy Type 1 group obtained from all 20 ROIs together using the SuStaIn algorithm. The analysis pipeline is illustrated in Fig. 1. For the 14 cortical ROIs based on thickness, the 95th percentile of the Z-scores was 3.5. Thus, we set the Z-score steps to 1, 2 and 3 with a Z-max of 4 for these regions. For the six ROIs utilizing volumes in the mesiotemporal and subcortical areas, the 95th percentile of the Z-scores was 2.9. Therefore, we set the Z-score steps to 1 and 2 with a Z-max of 3. In this setting, the SuStaIn sequence consists of 54 stages. To determine the maximum likelihood solution, computations were performed using 25 different starting points (random cluster assignments). Subsequently, an uncertainty estimation was conducted using 1 000 000 Markov Chain Monte Carlo samples initialized from the maximum likelihood solution. Computations were performed for up to three subtypes. Similar to previous studies, we performed a 10-fold cross-validation to assess the optimal number of subtypes and the consistency of subtype progression patterns.^25,34,35^ As outlined in a previous study, the optimal number of subtypes was determined based on information criteria calculated through cross-validation to avoid over-fitting.^25^ The consistency of subtype progression patterns was evaluated using cross-validation similarity (CVS), which averages the Bhattacharyya coefficients across all combinations of folds, measuring the similarity of Z-score events for corresponding subtypes (where zero indicates no similarity and one indicates maximum similarity). Subtype progression patterns identified by SuStaIn were visualized using BrainPainter.^42^

The subtype and stage of each individual were determined using SuStaIn. Each individual simultaneously expresses multiple subtypes, represented as probabilities ranging from zero to one, where the sum equals one. For the categorical classification, we adopted the subtype with the highest probability. Additionally, SuStaIn calculates the probability of each individual belonging to each stage. To determine the stage for each individual, as in previous studies, we calculated a weighted average by multiplying the probability of each stage by its stage number and summing these products.^34^ In this study, no individuals were assigned to stage 0 (i.e. with no abnormalities in thickness or volume in any region).

### Statistical analysis

We compared the age and MMSE scores between the control and myotonic dystrophy Type 1 groups using two-tailed Mann–Whitney and sex using χ^2^ tests. To assess differences between subtypes, we compared age and onset age among participants classified into each subtype using Mann–Whitney U-tests, and sex using χ^2^ tests. For other clinical indicators, we used an analysis of covariance (ANCOVA) controlling for age. Additionally, we compared the MMSE score, TIV, total ventricle volume and total brain parenchymal volume among all subtypes and the control group, controlled for age using ANCOVA. We applied false discovery rate (FDR) correction in the *post hoc* tests. We further examined the correlations of clinical indicators and volumetric measures with the probability of subcortical subtype (referred to as ‘subcortical probability’) using two-tailed Spearman rank correlation coefficients, controlling for age. As the probability of the cortical subtype is calculated as 1 – subcortical probability, these two probabilities are mathematically dependent. Therefore, in this study, we focused solely on the correlations with subcortical probability. We examined the correlations between the SuStaIn stage and each indicator using two-tailed Spearman rank correlation coefficients, controlling for age, except for age itself. Statistical significance was set at *P* < 0.05. All statistical analyses were performed using Python 3.8 (scipy version 1.7.3, statsmodels version 0.13.2 and pingouin version 0.5.4).

## Results

The clinical characteristics of the control and myotonic dystrophy Type 1 groups are presented in Table 1. The myotonic dystrophy Type 1 group comprised 60 individuals, whereas the control group included 50 individuals, with no significant differences in age or sex. The MMSE scores were significantly lower in the myotonic dystrophy Type 1 group (*U* = 1592.5, *P* < 0.001).

**Table 1:** Demographic characteristics of patients with myotonic dystrophy Type 1 and controls

	DM1 *n* = 60	Control *n* = 50	*P*
Age, median (range)	44 (20–67)	46.5 (17–66)	0.597
Onset age, median (range)	30 (7–61)		
Sex, F:M	32:28	28:22	0.930
CTG repeat length, median (range)	600 (100–2100)		
MIRS score, *n* (%)			
1	0		
2	16 (27)		
3	18 (30)		
4	18 (30)		
5	8 (13)		
MMSE, median (range)^a^	29 (13–30)	30 (26–30)	<0.001*

DM1, myotonic dystrophy Type 1; MIRS, muscular impairment rating scale; MMSE, mini-mental state examination.

^a^MMSE data were available for 40 patients.

^*^ *P* < 0.05.

### Two distinct subtypes identified by SuStaIn

**Figure 2:**
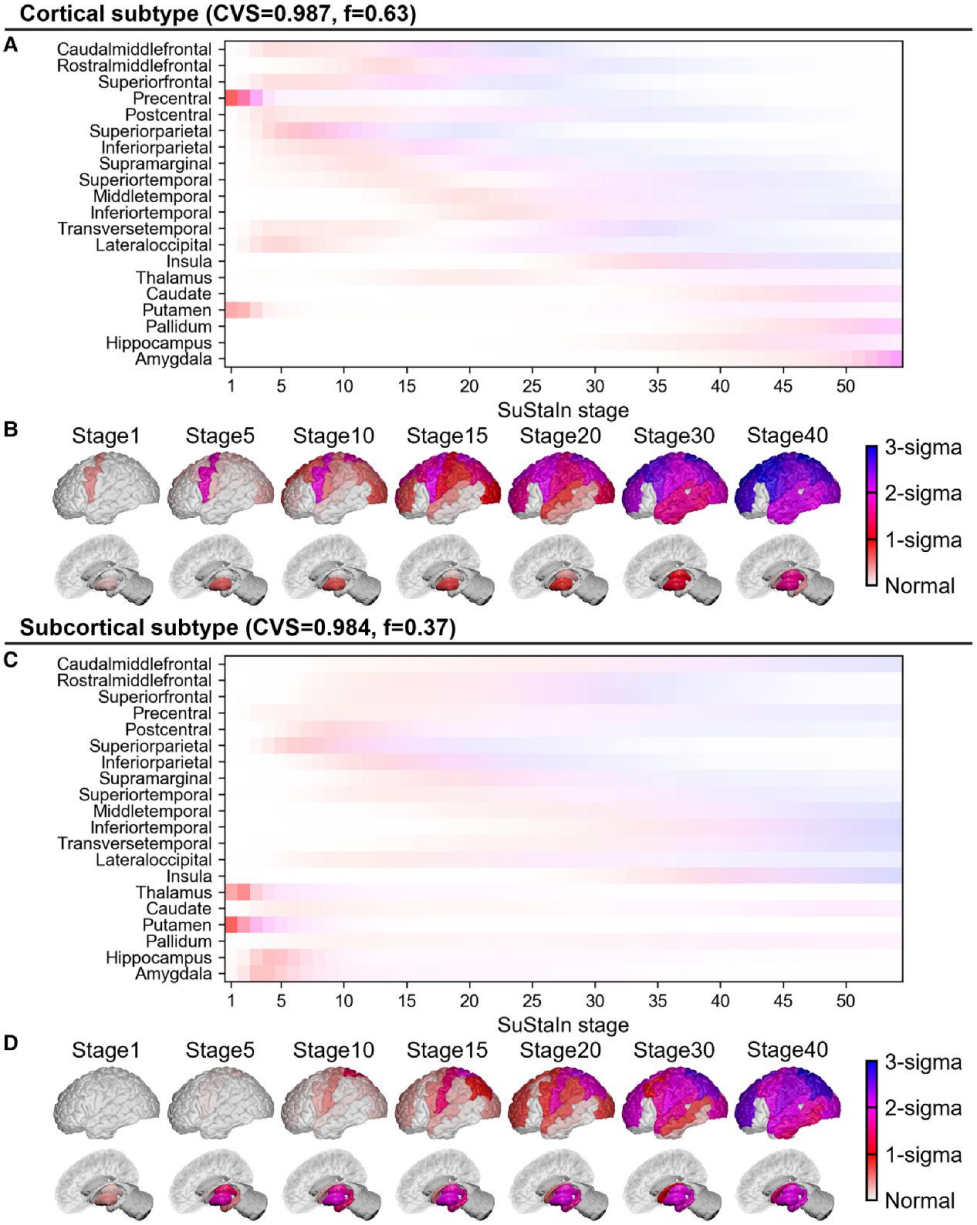
**Subtype progression patterns identified by SuStaIn. (A)** Uncertainty in the progression patterns of the cortical subtype, where each region is shaded according to the probability a particular Z-score is reached at a particular SuStaIn stage, ranging from zero (white) to one (red for a Z-score of one, magenta for a Z-score of two and blue for a Z-score of three). **(B)** Spatial distribution and degree of volume reduction at each SuStaIn stage of the cortical subtype. Colour shade represents the cumulative sum of probabilities in each brain region. **(C)** Uncertainty in the progression patterns of the subcortical subtype. **(D)** Spatial distribution and degree of volume reduction at each SuStaIn stage of the subcortical subtype. The “f” is a parameter of the SuStaIn model learned through model fitting that represents the proportion of each subtype. The “f” values shown in the figure were estimated through MCMC sampling. Visualizations in subfigures (B) and (D) were generated using BrainPainter. The anatomical regions described in this figure are labelled in Supplementary Fig. 2 for clarity. CVS, cross-validation similarity; MCMC, Markov Chain Monte Carlo; SuStaIn, subtype and stage inference.

SuStaIn identified two subtypes of brain involvement progression patterns in myotonic dystrophy Type 1 (Fig. 2). The cortical subtype, predominant in 39 individuals, showed a CVS of 0.987 (95% confidence interval [CI]: 0.986–0.988). In this subtype, volume reduction begins in the precentral gyrus and subsequently spreads to the frontal, parietal and occipital lobes. While an early volume reduction was observed in the putamen, other subcortical structures such as the hippocampus remained preserved until later stages. In contrast, the subcortical subtype, predominant in 21 individuals, exhibited a CVS of 0.984 (95% CI: 0.982–0.985). Volume reductions of the putamen, thalamus, hippocampus and amygdala progress from an early stage. Subsequently, cerebral atrophy gradually appeared; however, the atrophy of the precentral gyrus was not prominent. Progression patterns for each subtype across the cross-validation folds are shown in Supplementary Fig. 3.

### Clinical characterization of the subtypes

**Figure 3:**
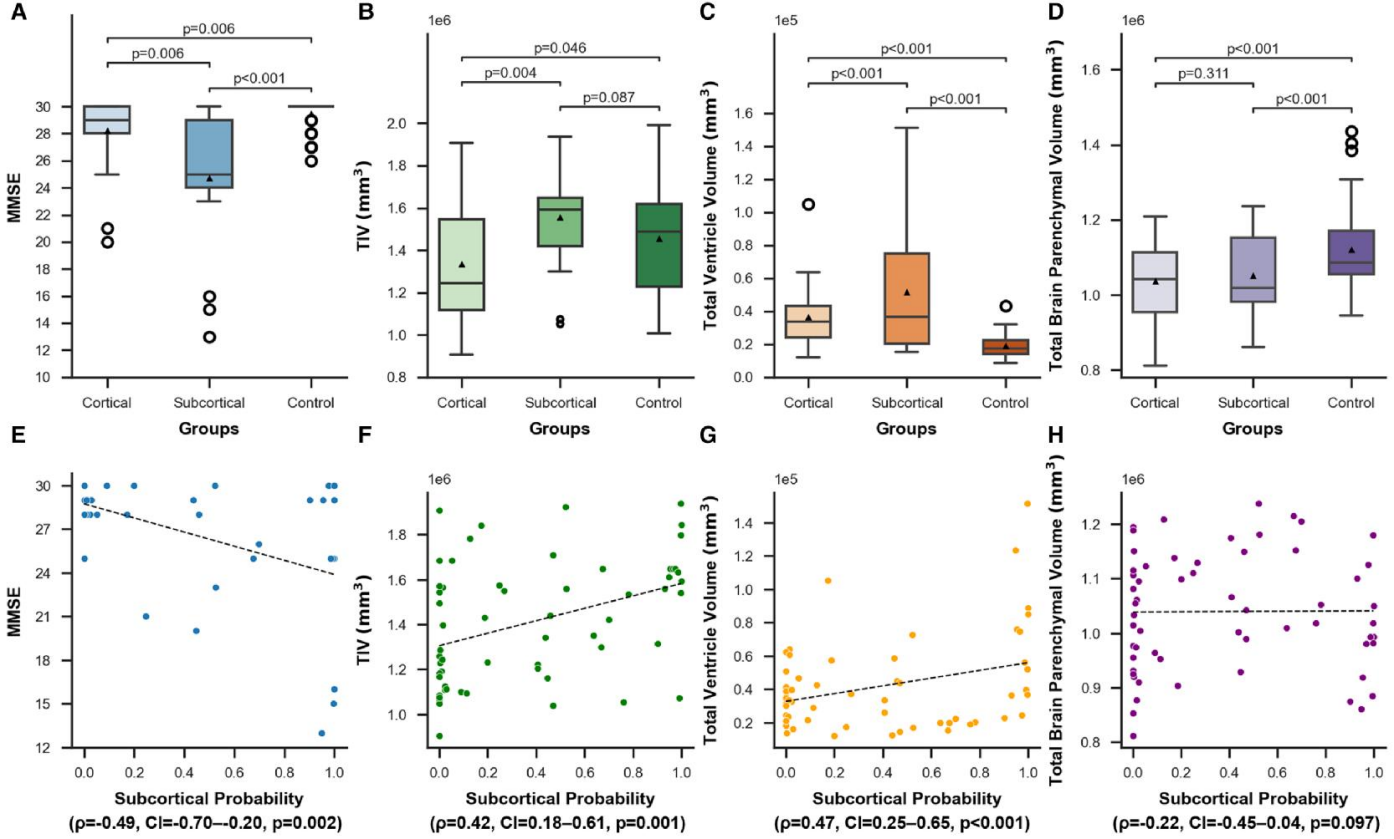
**Relationship between SuStaIn subtypes and MMSE score, TIV, total ventricle volume, and total brain parenchymal volume.** (**A–D**) Box plots showing the distribution of MMSE score, TIV, total ventricle volume and total brain parenchymal volume for each myotonic dystrophy Type 1 patient grouped by their highest probability subtype identified by SuStaIn. Comparisons included the control group for each metric. We compared each metric among all the subtypes and the control group, controlled for age using ANCOVA (*N* = 40 for MMSE scores and *N* = 60 for other metrics). We applied FDR correction in *post hoc* tests. Black triangles and black circles in the figure represent the mean values and the outliers, respectively. (**E–H**) Correlation between subcortical probability in myotonic dystrophy Type 1 patients and each metric. We examined the correlations using two-tailed Spearman rank correlation coefficients, controlling for age (*N* = 40 for MMSE scores and *N* = 60 for other metrics). MMSE showed a significant negative correlation (*ρ*=−0.49, CI = −0.70–−0.20, and *P* = 0.002), whereas TIV and total ventricle volume showed significant positive correlations (*ρ*=0.42, CI = 0.18–0.61, *P* = 0.001 and *ρ*=0.47, CI = 0.25–0.65, *P* < 0.001, respectively). Total brain parenchymal volume and subcortical probability were not correlated (*ρ*=−0.22, CI = −0.45–0.04, *P* = 0.097). MMSE, mini-mental state examination; SuStaIn, subtype and stage inference; TIV, total intracranial volume.

Next, we examined the differences in the clinical characteristics between the subtypes (Table 2). The age was significantly higher in the cortical subtype (median 47 years) compared to the subcortical subtype (median 41 years) (*U* = 573.0, *P* = 0.011); however, no significant differences were observed in onset age or sex (*U* = 513.0, *P* = 0.110/*χ^2^*[1] = 0.14, *P* = 0.704, respectively). When controlling for age, there were no significant differences in the MIRS scores (*F*(1,57) = 0.06, *P* = 0.800), repeat length (*F*(1,57) = 1.59, *P* = 0.213) and stage (*F*(1,57) = 0.96, *P* = 0.332). Significant differences in MMSE scores were observed among the three groups, including the control group (*F*(2,86) = 17.92, *P* < 0.001). Both subtypes exhibited significantly lower MMSE scores compared to the control group (*F*(1,72) = 8.60, FDR-adjusted *P* = 0.006 for the cortical subtype; *F*(1,62) = 8.60, FDR-adjusted *P* < 0.001 for the subcortical subtype). Furthermore, the subcortical subtype showed significantly lower MMSE scores than the cortical subtype (*F*(1,37) = 8.38, *P* = 0.006) (Fig. 3A). Additionally, we performed an analysis using both age and stage as covariates. The subtype differences remained unchanged, with MMSE scores being significantly lower in the subcortical subtype (*F*(1,36) = 7.39, *P* = 0.010). There were no significant differences in MIRS scores (*F*(1,56) = 0.16, *P* = 0.693) or repeat length (*F*(1,56) = 1.69, *P* = 0.199).

**Table 2:** Demographic characteristics of patients classified as cortical and subcortical subtypes

	Cortical *n* = 39	Subcortical *n* = 21	*P*
Age, median (range)	47 (24–67)	41 (20–60)	0.011*
Onset age, median (range)	30 (8–61)	27 (7–48)	0.110
Sex, F:M	17:22	11:10	0.704
CTG repeat length, median (range)	600 (100–1400)	600 (100–2100)	0.213
MIRS score, *n* (%)			0.800
1	0	0	
2	5 (13)	11 (52)	
3	16 (41)	2 (10)	
4	12 (31)	6 (29)	
5	6 (15)	2 (10)	
MMSE, median (range)^a^	29 (20–30)	25 (13–30)	0.006*
Stage, median (range)	14.3 (0.1–51.5)	4.9 (0.1–42.5)	0.332

DM1, myotonic dystrophy Type 1; MIRS, muscular impairment rating scale; MMSE, mini-mental state examination; SD, standard deviation.

^a^MMSE data were available for 40 patients (25 with cortical and 15 with subcortical subtypes).

^*^ *P* < 0.05.

We also compared TIV among the control, and cortical and subcortical subtypes, controlling for age. A significant difference was observed in TIV among the three groups (*F*(2106) = 5.88, *P* = 0.004). The TIV of the cortical subtype was significantly smaller than that of the control (*F*(1,86) = 4.81, FDR-adjusted *P* = 0.046) and the subcortical subtypes (*F*(1,57) = 11.53, FDR-adjusted *P* = 0.004). No significant difference was found between the subcortical subtype and control (*F*(1,68) = 3.02, FDR-adjusted *P* = 0.087) (Fig. 3B).

Owing to the differences in TIV among the groups, we compared the total ventricle volume and total brain parenchymal volume among the three groups using raw volumes instead of ratios to TIV. A significant difference was observed in the total ventricle volume among the three groups (*F*(2106) = 30.25, *P* < 0.001), with the subcortical subtype being significantly larger than the cortical subtype (*F*(1,57) = 14.98, FDR-adjusted *P* < 0.001). Additionally, the total ventricle volumes of both the cortical and subcortical subtypes were significantly larger than that of the control group (*F*(1,86) = 38.45, FDR-adjusted *P* < 0.001 and *F*(1,68) = 58.44, FDR-adjusted *P* < 0.001, respectively; Fig. 3C). A significant difference was also found in the total brain parenchymal volume among the three groups (*F*(2106) = 11.40, *P* < 0.001). Both the cortical and subcortical subtypes showed significantly smaller total brain parenchymal volumes than the control group (*F*(1,86) = 13.69, FDR-adjusted *P* < 0.001 and *F*(1,68) = 16.91, FDR-adjusted *P* < 0.001, respectively). However, no significant differences were observed between the subcortical and cortical subtypes (*F*(1,57) = 1.05, FDR-adjusted *P* = 0.311; Fig. 3D).

We examined the correlation between the subcortical probability and each indicator (Fig. 3E–H). As the subcortical probability increased, MMSE significantly decreased (*ρ*=−0.49, CI = −0.70–−0.20 and *P* = 0.002). Moreover, TIV and total ventricle volume positively correlated with the subcortical probability (*ρ*=0.42, CI = 0.18–0.61, *P* = 0.001 and *ρ*=0.47, CI = 0.25–0.65, *P* < 0.001, respectively). Total brain parenchymal volume and subcortical probability were not correlated (*ρ*=−0.22, CI = −0.45–0.04 and *P* = 0.097). There was no significant correlation between CTG repeat length or MIRS score and subcortical probability (*ρ*=0.04, CI = −0.21–0.30, *P* = 0.739 and *ρ*=0.05, CI = −0.21–0.30, *P* = 0.724, respectively).

### Clinical characterization of the stages

**Figure 4:**
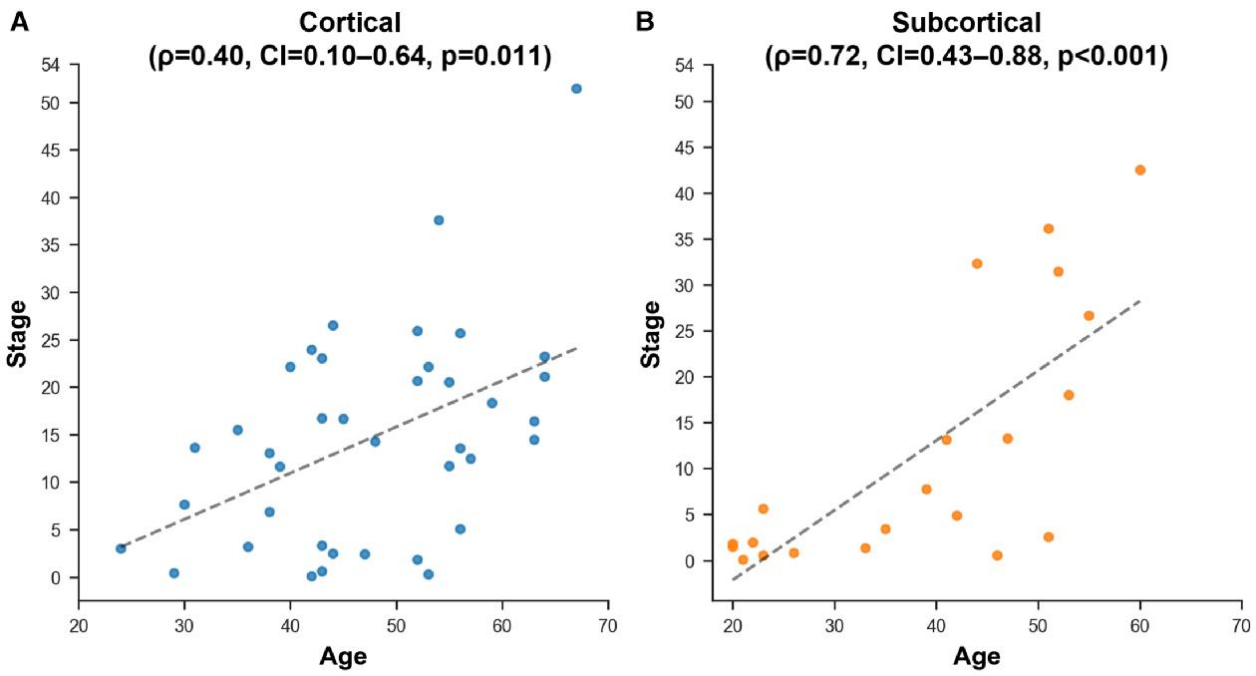
**Correlation between SuStaIn stage and age.** Both cortical **(A)** and subcortical **(B)** subtypes showed significant positive correlations between age and SuStaIn stage (*ρ*=0.40, CI = 0.10–0.64, *P* = 0.011 and *ρ*=0.72, CI = 0.43–0.88, *P* < 0.001). We examined the correlations using two-tailed Spearman rank correlation coefficients (*N* = 39 for the cortical subtype and *N* = 21 for the subcortical subtype). SuStaIn, subtype and stage inference.

We further examined the correlation between the stage and clinical characteristics. Both the cortical (*ρ*=0.40, CI = 0.10–0.64 and *P* = 0.011) and subcortical subtype (*ρ*=0.72, CI = 0.43–0.88 and *P* < 0.001) showed a correlation between age and stage (Fig. 4). Correlations with stage were not significant for onset age (*ρ*=−0.18, CI = −0.47–0.15 and *P* = 0.290 for the cortical subtype; *ρ*=0.05, CI = −0.40–0.48 and *P* = 0.843 for the subcortical subtype), CTG repeat length (*ρ*=−0.03, CI = −0.35–0.29 and *P* = 0.853 for the cortical subtype; *ρ*=0.114, CI = −0.35–0.53 and *P* = 0.632 for the subcortical subtype), MIRS score (*ρ*=0.19, CI = −0.14–0.48 and *P* = 0.261 for the cortical subtype; *ρ*=0.25, CI = −0.22–0.62 and *P* = 0.293 for the subcortical subtype) and MMSE (*ρ*=0.15, CI = −0.27–0.52 and *P* = 0.493 for the cortical subtype; *ρ*=−0.04, CI = −0.56–0.50 and *P* = 0.904 for the subcortical subtype).

## Discussion

We used SuStaIn on one of the largest datasets of patients with myotonic dystrophy Type 1 to elucidate the progression patterns of brain impairment in myotonic dystrophy Type 1. Two subtypes of myotonic dystrophy Type 1, cortical and subcortical, have been identified, with the subcortical subtype associated with cognitive decline. Furthermore, while the subcortical subtype showed similarly small brain parenchyma compared to the cortical subtype, significant enlargement of the brain ventricles and no significant decrease in TIV were observed, suggesting differences in early childhood brain development. This study demonstrates the potential of morphological MRI as a biomarker for cognitive impairment and brain developmental disorder in myotonic dystrophy Type 1.

The two subtypes identified using SuStaIn have distinct patterns of brain impairment progression. The cortical subtype showed volume reduction starting in the precentral gyrus, which spread to the frontal, parietal, and occipital lobes. Although the volume reduction of the putamen was observed in the early stages, other subcortical structures, including the hippocampus, were preserved until later. In contrast, during the early stages, the subcortical subtype showed volume reduction in the putamen, thalamus, hippocampus, and amygdala. Subsequently, cerebral atrophy gradually appeared; however, the atrophy of the precentral gyrus was not prominent. Previous studies using VBM have detected volume reductions in the frontal lobe, including the precentral gyrus, parietal and occipital lobes, putamen, thalamus, caudate nucleus and hippocampus.^11-13^ We used a large dataset for myotonic dystrophy Type 1 research, and the detected volume reduction patterns were consistent with those of previous studies; thus, it was considered to reflect the pathophysiology of myotonic dystrophy Type 1. Considering the progression trajectories and subtypes of volume reduction using SuStaIn, it was found that the atrophic regions shown in previous VBM studies were, in fact, the average of the atrophic regions of the two distinct subtypes.

In the subcortical subtype, the cognitive function assessed using the MMSE was significantly lower, and a significant negative correlation was found between subcortical probability and MMSE scores. In myotonic dystrophy Type 1, imaging findings on MRI, such as abnormal white matter signals do not serve as biomarkers of cognitive decline^43^; however, subcortical probability could become a useful biomarker. In clinical practice, large lateral ventricles observed in the subcortical subtype may indirectly serve as biomarkers. Furthermore, estimation of the subcortical probability may be possible by focusing on volume reductions specific to the subcortical subtype in the thalamus, pallidum, hippocampus and amygdala.

Cognitive decline in the subcortical subtype may be related to volume reductions in the hippocampus and amygdala. Weber *et al*. demonstrated that hippocampal atrophy in myotonic dystrophy Type 1 patients correlates with episodic memory deficits and clinical disease severity, suggesting that hippocampal pathology is a vital disease parameter in myotonic dystrophy Type 1.^20^ Furthermore, neuropathologically, neurofibrillary tangles accumulate in the cornu ammonis regions of the hippocampus, amygdala, and entorhinal cortex in myotonic dystrophy Type 1.^44^ Sergeant pathologically examined the spread of tau pathology in the brains of five myotonic dystrophy Type 1 patients.^45^ Tau pathology was observed in the hippocampus formation and amygdala in four out of five patients, of whom three had cognitive decline or mental retardation. Degeneration of the hippocampus and amygdala due to tau pathology is speculated to contribute to cognitive decline in myotonic dystrophy Type 1. The subcortical and cortical subtypes may correspond to the presence or absence of tau pathology in the hippocampus and amygdala.

In contrast, no significant difference was found in CTG repeat length or MIRS score between the subtypes. The CTG repeat length is measured using blood cells; however, it exhibits somatic instability, with the repeat length varying among organs and even within different brain regions.^2,45^ Although the relationship between the degree of CTG repeat expansion in each organ and the severity of impairment has not been concluded, it seems natural that there is no relationship between the subtype based on neurodegeneration and the repeat length measured in blood cells. Similarly, for the MIRS score, muscle degeneration and neurodegeneration were inferred to be independent. This interpretation is consistent with the absence of significant correlations between the stage, which reflects brain volume reduction, and CTG repeat length or MIRS scores.

As for the pathophysiology of the central nervous system in myotonic dystrophy Type 1, contributions not only from neurodegeneration but also from abnormal brain development are assumed.^8,9^ TIV is considered a marker of maximal brain growth and is generally determined by the age of 10.^9^ Several studies have reported that TIV is smaller in myotonic dystrophy Type 1, suggesting impaired brain development.^8,9,22^ In this study, the cortical subtype showed smaller TIV than controls, consistent with previous research, suggesting the involvement of early childhood brain development impairment. On the other hand, despite the subcortical subtype having total brain parenchymal volume similarly small as the cortical subtype, no significant difference in TIV was observed compared with controls. Additionally, the total ventricle volume was larger in both subtypes than in the controls, with the subcortical subtype showing greater enlargement. Two potential explanations for the lack of TIV reduction in the subcortical subtype are considered: first, early brain development is normal; second, early brain development is abnormal, but ventricular enlargement in childhood might contribute to larger cranial growth. In the first theory, preserved TIV in the subcortical subtype may be due to normal brain development until around the age of 10. In that situation, subsequent brain atrophy or pronounced ventricular enlargement might occur. In the second theory, brain volume is small from childhood; however, early ventricular enlargement might make the skull larger. Ventricular enlargement is known in adult-onset myotonic dystrophy Type 1 and has also been reported in congenital myotonic dystrophy Type 1.^46-49^ Considering this, it is possible that some patients with adult-onset myotonic dystrophy Type 1 show ventricular enlargement from childhood before symptoms appear. They may correspond to the subcortical subtype. In addition, regions showing volume reduction from an early SuStaIn stage might not have atrophied but could instead be small due to developmental impairment. However, as no studies have examined the head and brain morphology during childhood in adult-onset myotonic dystrophy Type 1, both theories remain speculative.

A positive correlation was noted between the SuStaIn stage and age. Previous studies, including longitudinal studies, have also shown that brain atrophy progresses with ageing in myotonic dystrophy Type 1, consistent with our findings.^14-16,40^ Myotonic dystrophy Type 1 has aspects of both RNAopathy and spliceopathy. More specifically, expanded CTG repeats in the non-coding regions of the *DMPK* gene lead to the accumulation of mutated RNA in the nucleus, where the resulting nuclear foci sequester RNA-binding proteins and disrupt RNA metabolism and splicing.^2^ Simultaneously, myotonic dystrophy Type 1 is also a tauopathy characterized by the accumulation of neurofibrillary tangles in the brain.^2^ The progression of age-related brain volume loss in myotonic dystrophy Type 1 is presumed to be associated with the gradual accumulation of mutated RNA and tau over time. However, as no significant correlation was observed between the stage and MMSE score, the impact of age-related brain volume reduction on cognitive function may be limited. Abnormal brain development driven by genetic factors may play a role in the cognitive function of patients with myotonic dystrophy Type 1.

This study has several limitations. First, this was a single-centre study without an external validation cohort. However, the distribution of the volume reduction observed in this study aligns with that of previous VBM studies on myotonic dystrophy Type 1, suggesting that our findings generally reflect the pathology of myotonic dystrophy Type 1. Additionally, previous studies have consistently demonstrated the high reproducibility of the SuStaIn across independent datasets.^25,34,35^ Second, only a subset of brain regions was used in the analysis. Owing to computational complexity, the number of ROIs in the SuStaIn analysis is limited. We selected and analysed 20 ROIs based on the regions of volume reduction identified in previous VBM studies to retain as much information as possible about the brain features of myotonic dystrophy Type 1. Third, obtaining the ground truth to assess the accuracy of the trajectories estimated using the SuStaIn algorithm is difficult. Although it may be possible to confirm whether the longitudinal data follow the identified disease progression patterns, this must be verified in future studies. Fourth, as in previous studies, we used linear regression to control for confounding factors such as age when calculating Z-scores. However, a nonlinear approach, such as generalized additive models, may have better captured complex relationships between confounding factors and brain morphology.

In conclusion, we identified two trajectories of brain volume loss, cortical and subcortical, in myotonic dystrophy Type 1 using SuStaIn. In the cortical subtype, volume loss begins in the precentral gyrus and primarily spreads to the cerebral cortex. In the subcortical subtype, the volume loss progresses from the putamen, thalamus, hippocampus and amygdala. We also found that the subcortical subtype was associated with cognitive decline. Additionally, cranial and ventricular volumes in the subcortical subtype were larger than those in the cortical subtype, suggesting differences in trajectories from childhood. Machine learning can generate new insights that may aid in the clinical stratification of myotonic dystrophy Type 1 and contribute to a better understanding of its complex pathophysiology.

## Supplementary Material

fcaf181_Supplementary_Data

## Data Availability

Anonymized statistical data to reproduce the main findings are available from the corresponding author upon reasonable request from a qualified investigator. The Python implementation of the SuStaIn algorithm is available at https://github.com/ucl-pond. BrainPainter is available at https://github.com/razvanmarinescu/brain-coloring.
