# Supplementary material for: Two distinct trajectories of brain volume loss in myotonic dystrophy type 1 via machine learning
Source: Brain Commun. 2025 May 7;7(3):fcaf181. doi: 10.1093/braincomms/fcaf181 (PMC12094019; doi:10.1093/braincomms/fcaf181)
Supplement: fcaf181_Supplementary_Data [file fcaf181_supplementary_data.pdf]

Supplementary Material

Two Distinct Trajectories of Brain Volume Loss in Myotonic Dystrophy Type 1 via Machine Learning

Supplementary Figure

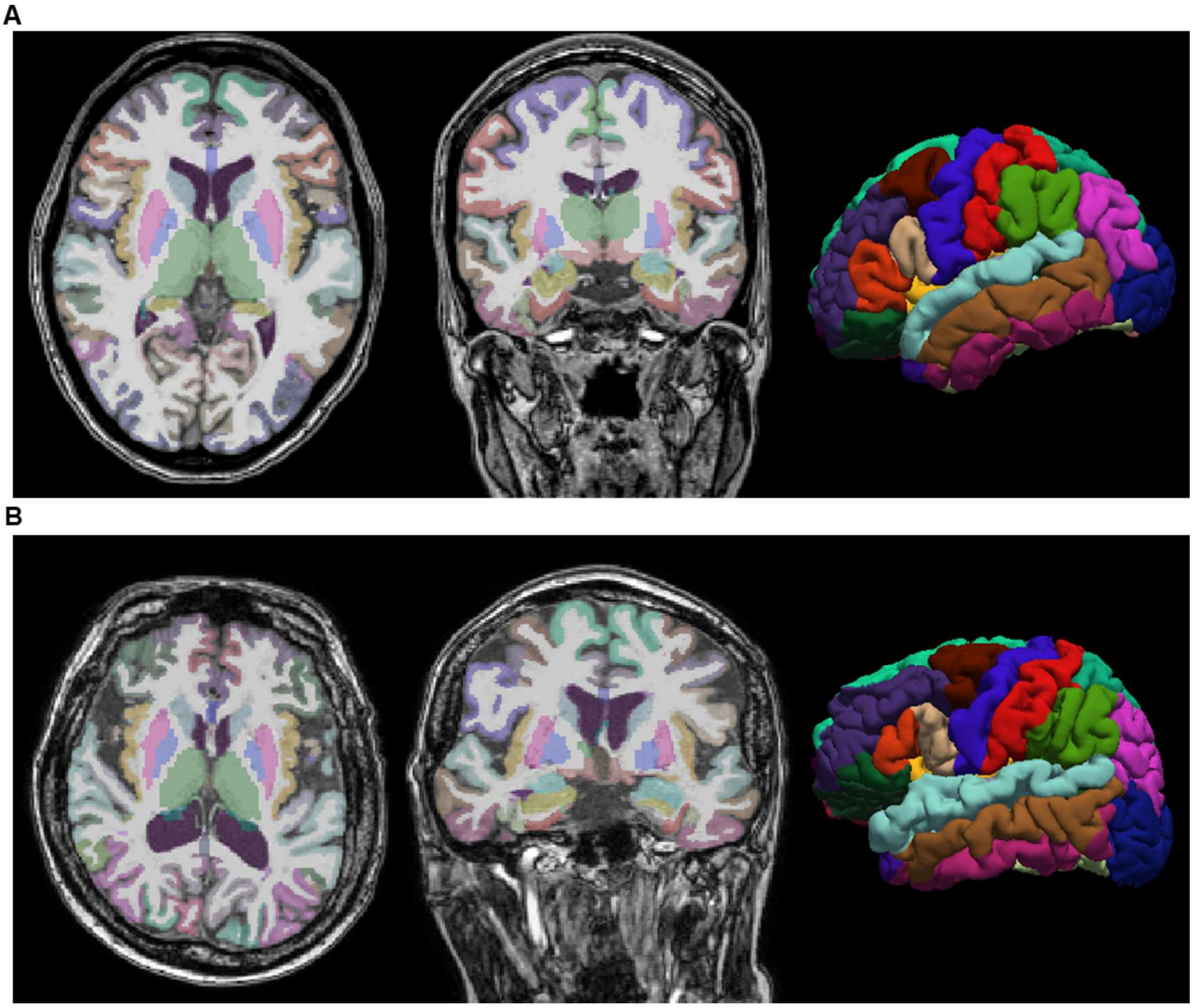

Supplementary Figure 1 Segmentation results with the highest and lowest Euler numbers. Using FreeSurfer 7.4 (http://surfer.nmr.mgh.harvard.edu/), we segmented 3D T1-weighted images for all participants. In addition to visual inspection, we assessed the quality of cortical surface reconstructions using the Euler number. For a completely flat and smooth surface, the Euler number should be 2. For a surface containing defects, the Euler number is

calculated as 2−2n, where n is the number of defects. **(A)** The segmentation result with the highest Euler number (=-16) is presented. **(B)** The segmentation result with the lowest Euler number (=-204) is presented. Even the lowest Euler number falls within the acceptable range, and visual inspection confirms that the segmentation quality is sufficient. From left to right, segmentation results in axial slice, coronal slice, and brain surface are shown.

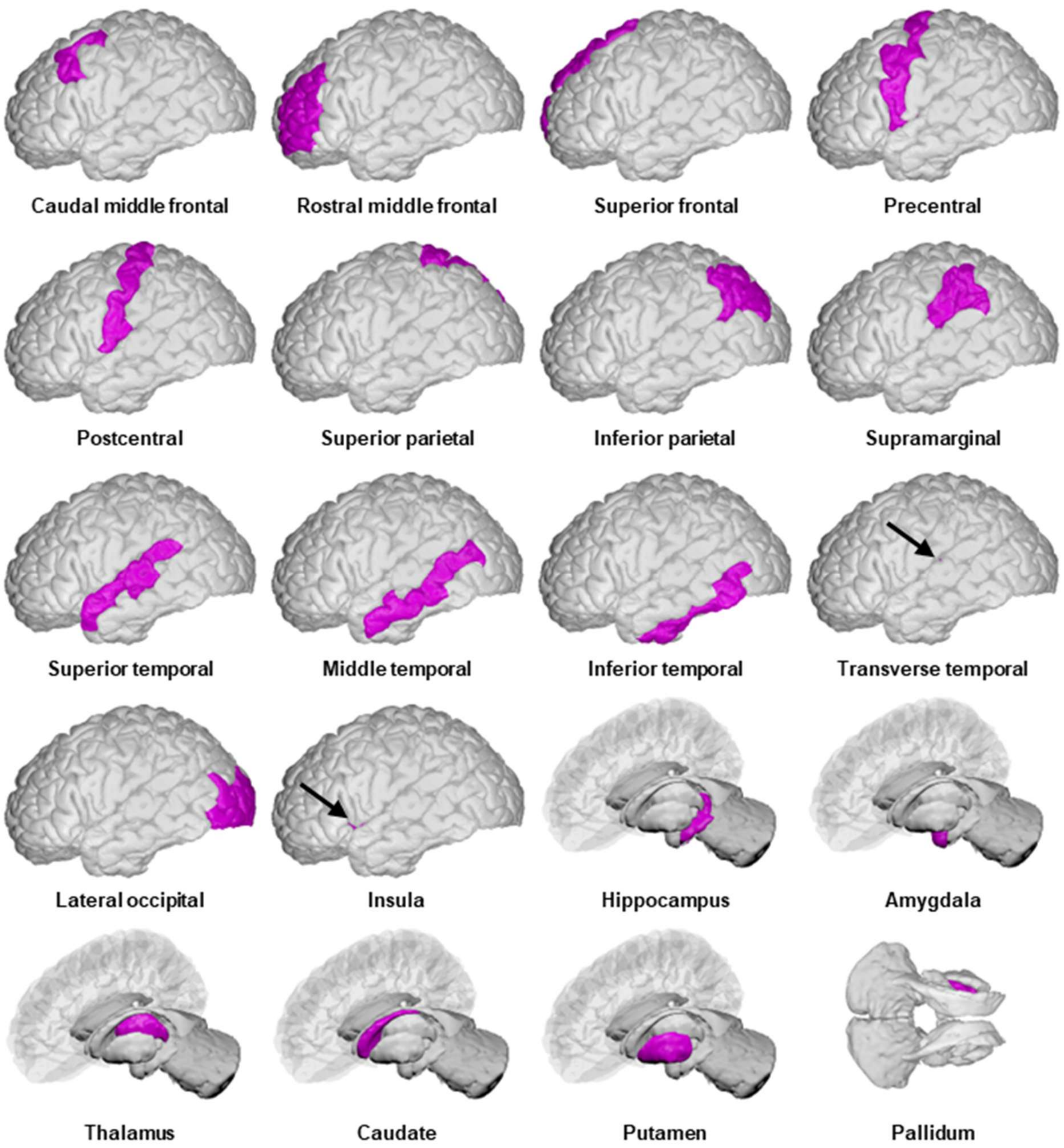

Supplementary Figure 2 Region of interest for SuStaIn. We selected 20 ROIs: the caudal middle frontal, rostral middle frontal, superior frontal, precentral, postcentral, superior parietal, inferior parietal, supramarginal, superior temporal, middle temporal, inferior temporal, transverse temporal, lateral occipital, insula, thalamus, caudate, putamen, pallidum, hippocampus, and amygdala. Cortical ROIs were based on the Desikan–Killiany Atlas, whereas subcortical ROIs were based on an atlas containing probabilistic information regarding the location of structures. These regions correspond to those illustrated in Fig. 2. ROI, region of interest.

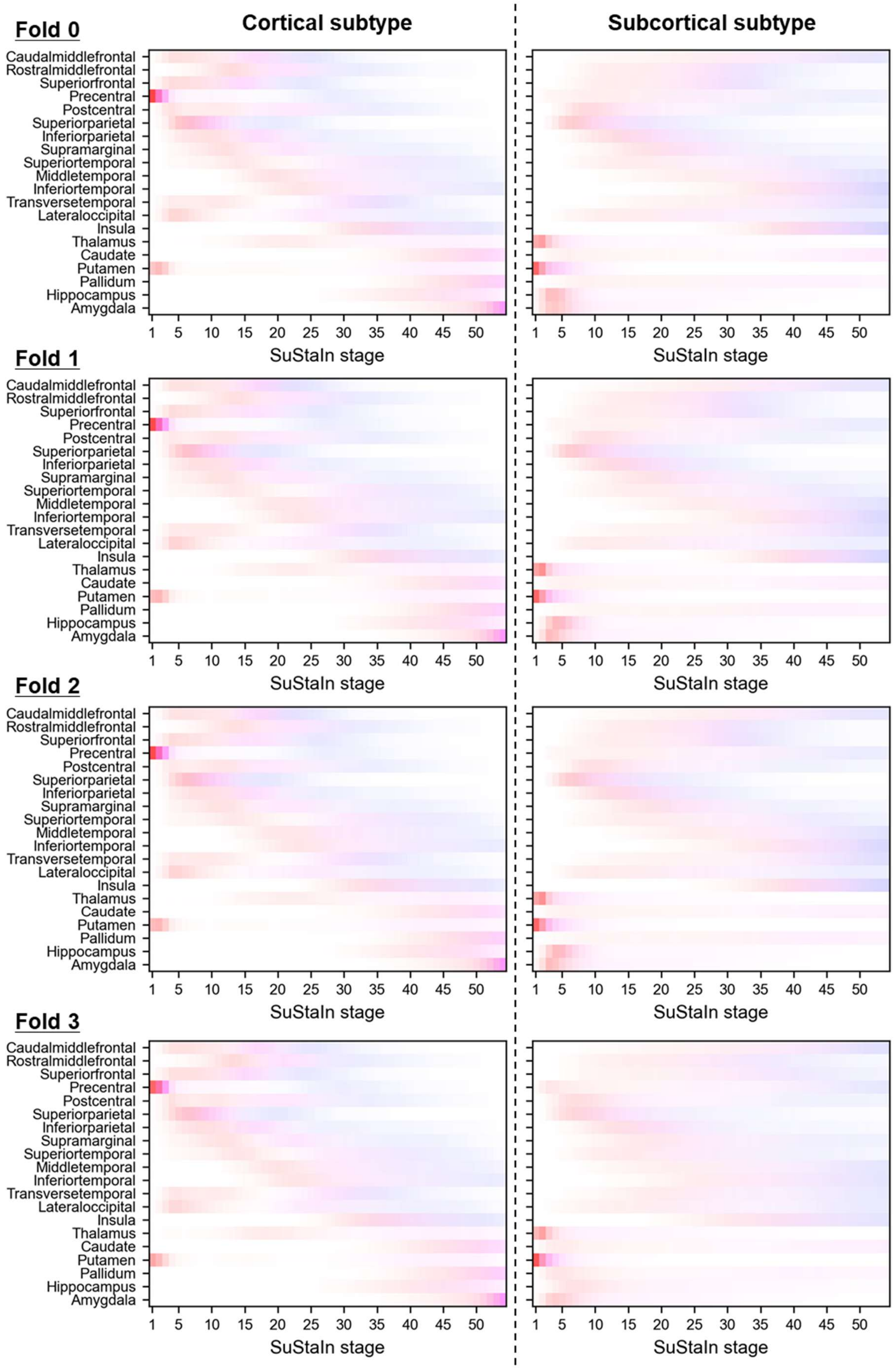

Fold 0
Cortical subtype
Subcortical subtype
Caudalmiddlefrontal
Rostralmiddlefrontal
Superiorfrontal
Precentral
Postcentral
Superiorparietal
Inferiorparietal
Supramarginal
Superiortemporal
Middletemporal
Inferiortemporal
Transversetemporal
Lateraloccipital
Insula
Thalamus
Caudate
Putamen
Pallidum
Hippocampus
Amygdala
1 5 10 15 20 25 30 35 40 45 50
SuStaIn stage
Fold 1
Fold 2
Fold 3

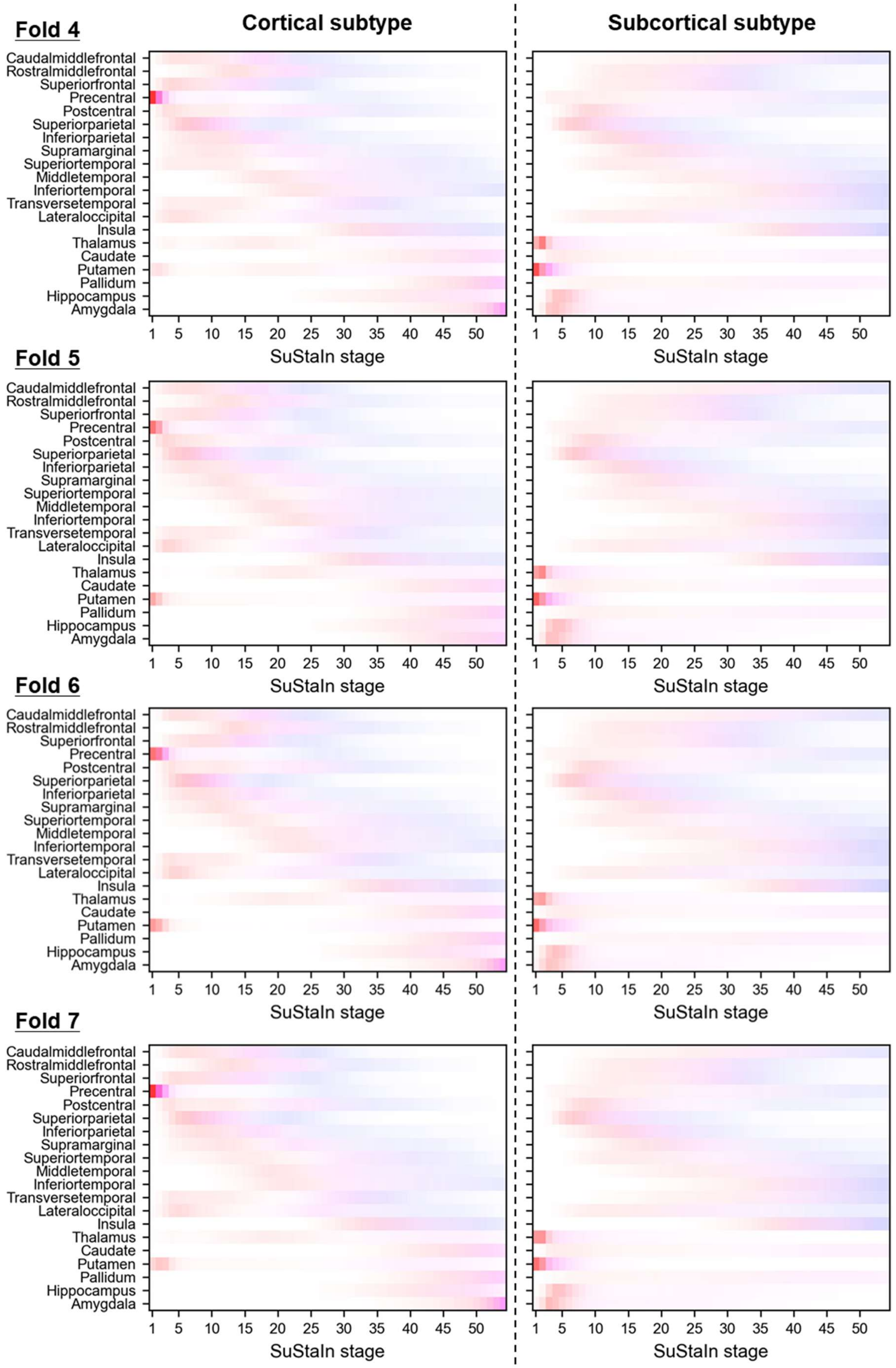

Fold 4
Cortical subtype
Subcortical subtype
Caudalmiddlefrontal
Rostralmiddlefrontal
Superiorfrontal
Precentral
Postcentral
Superiorparietal
Inferiorparietal
Supramarginal
Superiortemporal
Middletemporal
Inferiortemporal
Transversetemporal
Lateraloccipital
Insula
Thalamus
Caudate
Putamen
Pallidum
Hippocampus
Amygdala
1 5 10 15 20 25 30 35 40 45 50
SuStaIn stage
Fold 5
Fold 6
Fold 7

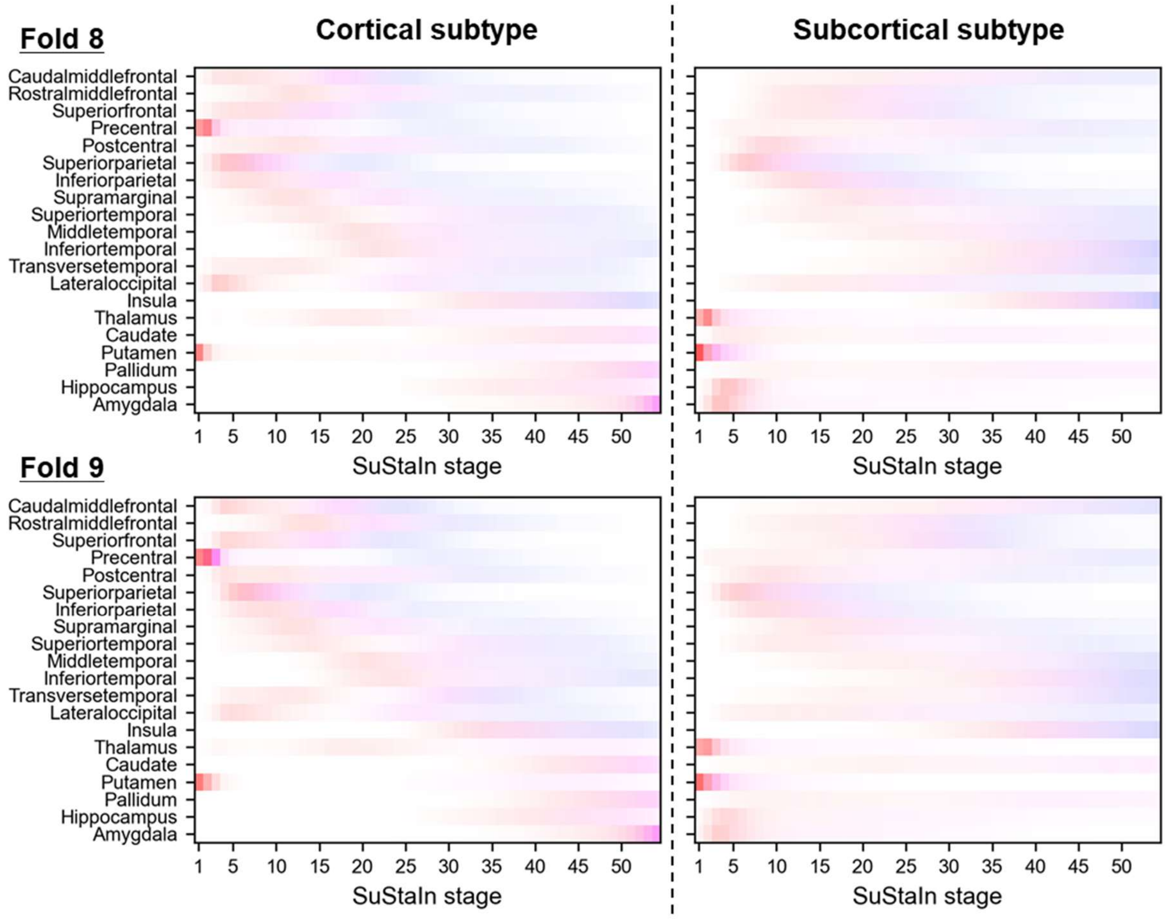

Supplementary Figure 3 Reproducibility of subtype progression patterns under cross-validation. Subtype progression patterns for each subtype across cross-validation folds are shown. Each region is shaded based on the probability of reaching a specific Z-score at a given SuStaIn stage, ranging from zero (white) to one (red for a Z-score of one, magenta for a Z-score of two, and blue for a Z-score of three). High reproducibility of subtype progression patterns was confirmed, with CVS of 0.987 (95% CI: 0.986–0.988) for the cortical subtype and 0.984 (95% CI: 0.982–0.985) for the subcortical subtype. CI, confidence interval; CVS, cross-validation similarity; SuStaIn, subtype and stage inference.
